# The impact of COVID-19 on supply decision-makers: the case of personal protective equipment in Spanish hospitals

**DOI:** 10.1186/s12913-021-07202-9

**Published:** 2021-10-28

**Authors:** Manuel F. Morales-Contreras, Marcelo Leporati, Luciano Fratocchi

**Affiliations:** 1 Faculty of Business Management and Economics, ICADE, Universidad Pontificia Comillas, Madrid, Spain; 2 Institute for Research in Technology (IIT), ICAI School of Engineering, Universidad Pontificia Comillas, Madrid, Spain; 3 EAE Business School, Madrid, Spain; 4 University of L’Aquila, L’Aquila, Italy

**Keywords:** Health care, Hospital, COVID-19, Personal protective equipment (PPE), supplies, Purchasing.

## Abstract

**Background:**

The COVID-19 pandemic has been recognized as a trigger for redefining supply chains at the global level, and has created an intense debate within the academic community and among policy-makers and practitioners. Among other industries, health care has been dramatically hit by the scarcity of “medical products,” specifically for personal protective equipment (PPE-like), due to supply chain disruptions coupled with dramatically increased demand. We aimed to analyze how the scarcity of PPE-like during the COVID-19 pandemic has modified the behavior of decision-makers in the PPE-like supply chain at the hospital level, and to explore what changes could be implemented to cope with future PPE-like shortages.

**Methods:**

We used an explorative approach based on semi-structured interviews with key informants in the Spanish health care industry. More specifically, we held interviews to industry experts at three hospitals in three Spanish regions to map the consequences of the COVID-19 pandemic onto the buying decision-making process.

**Results:**

Different strategies were developed by decision-makers at hospitals before, during, and after the first wave of the COVID-19 pandemic in Spain. Our paper offers two main findings: a) decision-makers changed their purchasing behavior from a cost main driver to guaranteeing the availability of supplies; b) they supported the idea of giving more “strategic autonomy” to Spain or Europe through back and nearshoring decisions.

**Conclusions:**

This paper could be of interest to health care management at the national, regional, and hospital levels, as well as for policy-makers, since it could help to establish and configure policies to support the sourcing of medical products (specifically PPE-like) to anticipate potential supply disruptions. Our paper contributes to the limited existing literature on how purchasing strategies at the decision-maker level and supply vary in the health care industry when a public health crisis appears, and what potential solutions might be for policy-makers and practitioners involved in the health care industry.

## Introduction

The World Health Organization (WHO) declared the COVID-19 pandemic on March 11, 2020; more than 110,000 cases and more than 4000 deaths had been reported in more than 110 countries [1]. The COVID-19 pandemic has caused tremendous disruption in the global supply chains of almost every sector. In particular, the supply of medical products became a critical element for every country, as they were needed to manage and control the evolution of the pandemic [2, 3]. By “medical products,” experts generally mean a wide range of product categories related to equipment used in the health care industry that are different from drugs [4]. These product categories range from simple products (primarily represented by personal protective equipment, or PPE-like) to high-tech products (e.g., ventilators, magnetic resonance imaging equipment, etc.). Technical and technological differences among different product lines induce division of the “medical products” sector into two different business areas: PPE-like and medical devices [5]. In this paper, we focus on the first category (PPE-like), including face masks (medical masks and N95-type respirators) and gloves. Both product lines have witnessed a dramatic surge in demand since the beginning of the pandemic, resulting in a huge scarcity within the health care system [3]. These product lines have experienced some intensive waves of offshoring strategies implemented in recent decades, which made the face mask and gloves supply chain very long and complex, involving many manufacturers, distributors, and other intermediaries [6]. More specifically, before the COVID-19 pandemic, most PPE-like stock was manufactured in China [7], with the US being the world’s largest importer of many of them, such as face masks, eye protection, or medical gloves [8]. The offshoring strategy implemented in the PPE-like industry was mostly carried out due to economic issues; in this regard, it is worth noting that cost optimization and minimizing inventories had been a priority for health care systems for a long time [6, 7, 9].

In the early stages of the COVID-19 crisis, China became the central customer for PPE-like factories. However, when the pandemic reached the rest of the world, all national governments had to manage the critical activity of finding PPE-like (and especially face masks) [3]. Countries, cities, federal states/regions, hospitals, and other health-related institutions were all competing for the same limited supply of face masks, creating a public health and national security crisis in every country [10]. Due to the scarcity of PPE-like during the pandemic, health care professionals were not provided with proper protective equipment. They admitted fear in different ways, such as becoming infected themselves, their family members becoming infected, or becoming a carrier for rapid spread of the disease [11, 12].

Highly dependent on imports, some countries such as the US [8] or Spain [13] became highly vulnerable to disruptions of medical supplies [3]. There was panic in some governments, pushing them to implement actions described as “modern piracy,” such as in the case of confiscating PPE-like orders initially allocated to other countries. For instance, US officials blocked German orders, while France and Germany decided to block exports to Spain and Italy [7].

Therefore, delivery times to Europe and prices rose significantly during the pandemic [14]; moreover, new regulations were implemented in China for the local market, which differed from those in the international market (CE certificate, stricter quality control, and more rigorous inspection rates). Additionally, research conducted in the US showed that emergency use authorizations were allowed to source new references from new suppliers, mostly from China and Europe. As a result, not all of them complied with the required standards and quality parameters [15].

Spain was one of the European countries most affected by the pandemic, especially during the first wave, with one of the highest death rates per capita, one of the largest health worker infection rates, and one of the most intensive, extensive lockdowns in the world [16, 17]. As noted by Blanco-Donoso et al. [13] “the lack of material resources (i.e., PPE-like) and sufficient personnel to deal with the emergency health situation caused by this virus has contributed to this situation.” In addition, the federal organization of the Spanish health care system generated criticalities.

Similar to previous public health crises [6], COVID-19 may also represent a source of lessons for decision-makers at different levels of the health care system (e.g., national and regional governments and hospital management). Therefore, we examined how COVID-19 impacted the Spanish health care system; in so doing, we shed new light on the following two research questions:

- How has the scarcity of PPE-like during the pandemic modified the behavior of hospital supply decision-makers in Spain?
- What changes could be implemented to cope with future PPE-like supply shortages?

To fulfill the proposed research objectives, we designed an explorative, qualitative methodology to answer these questions. We held semi-structured interviews with different managers involved in the purchasing process within public health institutions in Spain.

The article is structured as follows: Section 2 reviews the theoretical framework regarding the pandemic’s impact on medical products as well as the health care system in Spain. Section 3 describes the justification and design of the methodology. Section 4 presents the findings. The final sections include the discussion and conclusions.

## Literature review

### The pandemic’s impact on PPE-like availability during the COVID-19 crisis: evidence and lessons for the future

To define a comprehensive theoretical framework, in this section, we will summarize three key issues. First, we will define the global value chain (GVC) of “medical products” to describe its peculiarity. Next, we will describe empirical evidence of the consequences of COVID-19 on the supply of PPE-like agents, both in the European Union (EU) and Spain. Finally, we will explore possible solutions found in the current literature.

#### The “medical products” GVC

The European “medical products” market (EU27 plus Norway, Switzerland, and the UK) accounted for approximately 27% of the global market and has grown at an annual average rate of 4.2% in the past decade, with Germany (27.1%), France (14.6%), and the UK (11%) being the largest countries [18]. European countries’ dependence on imports of “medical products” as a whole is still relevant, reaching up to 1% of all imports (as in the case of Belgium and the Netherlands). Within these imports, the percentage of products coming from China, regarding total values, ranges from a minimum of 0.85% (Belgium) to a maximum of 15% (Malta), with Spain at 3.59% [16].

The distinction of “medical products” between “PPE-like” and “medical devices” [5] is also pertinent to GVC structures. While PPE-like product lines were massively offshored and outsourced to lower-cost countries, the “medical devices” GVC is dominated by a small number of EU and US multinationals [19]. For example, the production of examination gloves is almost entirely located in Malaysia [20]. At least before the COVID-19 pandemic, almost half of the world’s face mask production was concentrated in China [21]; another significant percentage came from Turkey. In contrast, approximately 60% of the overall ventilator market is owned by three European firms [19], even if their suppliers are scattered around the world [22]. While considering the structure of the GVCs for the “PPE-like” and “medical devices” product lines, several differences emerged (Table 1); the only similarity regards the increased role of imports from China.

**Table 1:** Differences among GVCs according to product subcategories

	Medical devices	PPE-like
**Type of GVC**	Driven by EU and US producers	Market-driven
**Critical success factor**	Respect for technical standards (even if cost pressures by national health systems have mounted in recent years)	Costs
**Degree of production offshoring**	Low (due to regulatory issues)	High
**Degree of production outsourcing**	Low (due to regulatory issues)	High
**Distribution channel**	Direct relationships with health care industry buyers (e.g., hospitals)	Wholesale distributors
**EU countries’ dependence on Chinese imports (Jan/Jul 2019 vs. 2020)**	7–30%	44–76%

Source: Our elaboration is based on the Directorate General for External Policies of the Union [5]

Among PPE-like, facial masks are used to prevent contagion, as well as the spread of respiratory infections [3]. More specifically, they provide different levels of protection according to their ability to filter the air that the user breathes; the N95 type is the highest performing.

Facial masks are basic products and cheap, but their manufacturing involves several types of inputs; therefore, the production process is fairly sophisticated [3, 23]. The production of N95 masks requires special fabrics and very strict quality control to guarantee functional properties, such as the ability to filter out small particles, a tight fit to the face, and low insulation resistance [15]. Buying organizations in the health care industry (hospitals and health institutions) generally do not have the capability to perform filtration tests on face masks. These tests are normally performed by the manufacturer in-house or by an outsourced specialized laboratory. This makes adherence to standards a key trust issue for PPE-like firms [24].

#### PPE-like shortage during the COVID-19 crisis: evidence from Europe and Spain

As recently noted, “before the COVID-19 pandemic, the production and supply chains of medical products did not attract much attention, largely because markets for most of these products worked smoothly and delays for shortages were the exception rather than the rule” [5]. After the first quarter of 2020, huge difficulties emerged in providing a sufficient supply of critical medical products, indirectly confirming the expectation of Shingal and Agarwal [25] that the COVID-19 pandemic would be likely to have consequences on GVCs; this is more relevant than the previous SARS and MERS pandemics, and also because of the wider impact at the global level.

As far as empirical evidence goes, data from Eurostat show that in comparing January–July 2019 with the same period in 2020, PPE-like demand surged rapidly around the world, obliging countries to depend heavily on imports from Asian countries. In Europe, for instance, imports of protective garments and similar products increased by 145%, while those of facemasks rose by 1.462% (from 1.1 billion to 17.2 billion). Moreover, prices skyrocketed with surgical masks from China, increasing by 182% from February to March 2020 [26]. Such evidence is mostly related to both the concentration of production in a single country (e.g., inspection gloves in Malaysia) and to the unavailability of inputs (e.g., melt blown, non-woven fabric for facial masks) due to export bans, logistic network interruptions, and shortages of packaging [3, 27]. Moreover, due to pandemic’s origin in China, exports from that country immediately came to a halt at the very beginning of 2020, when COVID-19 started to enter European countries (starting from Italy at the end of February).

#### Proposals for coping with PPE-like scarcity in the literature

Given the critical role of PPE-like during public health emergencies, some authors have already investigated the risk characterizing the relevant supply chains. In this regard, Patel et al. [6] pointed out that “the US PPE-like supply chain has minimal ability to rapidly surge production, resulting in challenges to meet large unexpected increases in demand that might occur during a public health emergency”. This evidence is then coupled with the significant proportion of the PPE-like supply chain located offshore, making the risk of scarcity extremely high, as in the case of the 2009 H1N1 influenza pandemic and the 2014 Ebola virus epidemic. Based on such a public health emergency, the authors developed some useful suggestions, such as the idea of monitoring PPE-like use and distribution to minimize inappropriate purchases, and to improve overall PPE-like distribution across the health care system. At the same time, suppliers’ sharing among different organizations that have requested PPE-like should be incentivized. Moreover, information sharing and regular communication flows between the private sector and local/national government authorities would be extremely useful. Finally, more elasticity should be ensured for the distribution system in implementing stockpiles (see also [8]), either in health care facilities, at the distributor level, at the manufacturer level, or by federal and/or state public health agencies.

Hackenborich et al. [16] suggested coupling subsides for production reconversion with regulatory adjustments to speed up certification processes for new products (e.g., simplified ventilators).

More recently, the role of costs in the purchasing decision-making process has been pointed out by previous studies. For instance, Sharma et al. [28] analyzed COVID-19’s impact on the supply chain decision-making process, and underscored that “a decades-long focus on supply chain optimization to minimize costs, reduce inventories, and drive up asset utilization has removed buffers and flexibility to absorb delays and disruptions”. At the same time, Cohen and Rodgers [8] found that “market prices are not appropriate mechanisms for rationing inputs to health because health is a public good”.

To cope with the scarcity of medical products (especially during the first wave of the pandemic in the first quarter of 2020) and to handle future pandemics, several strategies were proposed and at least partially implemented. From a very short time perspective, the US Centers for Disease Control and Prevention recommended using N95 respirator masks only during aerosol-generating procedures. Moreover, hospital staff received guidelines suggesting reusing masks intended for one-time use, and even scarves and bandanas in the case of facial mask stocks being fully depleted [21]. At the same time, some changes were adopted in public procurement strategies, introducing, for instance, joint procurement at the national level to gain bargaining power when contracting with foreign producers and intermediators [5]. In this respect, it is worth noting the European Commission’s decision to create a strategic stockpile of medical equipment (e.g., intensive care medical equipment such as ventilators, PPE-like equipment such as reusable masks, vaccines and therapeutics, laboratory supplies) such as ventilators and protective masks to help EU countries in the context of the COVID-19 pandemic [29]. Grumiller and Grohs [19] pointed out that stockpiling PPE-like products is easy but requires coordination at the EU level, which is hard to obtain from a long-term perspective, with the health care system being a prerogative of national countries [16].

Further, diplomatic efforts were implemented to prevent export bans. Some national governments also activated subsidies for new production lines for final products (e.g., facial masks) and inputs (e.g., melt blown) [16]. Several companies (especially in the textile and garment industries) reconverted their production lines to make facial masks and other PPE-like products [30, 31]. At the same time, wine and spirits makers partially reconverted their production capacity to produce disinfectants both in Spain [32–35] and other European countries, such as Italy [36]. Other interesting collaborations took place in the ventilator business, where the dramatic rise in demand was managed by partnerships with firms from other sectors, such as in the case of Ferrari automakers in Italy [37], SEAT automakers in Spain [38], and/or technical universities [39].

From a medium-term perspective, Cohen and Rodgers [8] proposed a list of measures that could secure the supply of PPE-like in the future: “removing the profit motive for purchasing PPE-like in hospital costing models, strengthening government capacity to maintain and distribute stockpiles, developing and enforcing regulations, and pursuing strategic industrial policies to reduce US dependence on imported PPE-like”.

Regarding Western countries’ dependence on imported PPE-like, COVID-19 triggered a renewed debate on the opportunity to carry out reshoring strategies to ensure that European countries would have an adequate level of “strategic autonomy” [5, 40]. General label reshoring is adopted to refer to a “a voluntary corporate strategy regarding the home country’s partial or total relocation of (in-sourced or out-sourced) production to serve local, regional, or global demand” [41]. More specifically, such a strategy may be executed to relocate production activities to the home country (so-called “back-shoring”), the home region (“nearshoring”), and a new foreign host country far away (“further offshoring”) [41]. Recently, UNCTAD [42] proposed four trajectories of international production after the COVID-19 pandemic: diversification, replication, reshoring (referring to the back-shoring alternative), and regionalization (i.e., the near-shoring alternative). In this regard, Barbieri et al. [40] found that the COVID-19 pandemic may act as a trigger for both back-shoring and near-shoring. This is consistent with the call of the WHO’s General Manager, Tedros Adhanom Ghebreyesus, to governments to increase domestic manufacturing of PPE-like products [7]. At the same time, back-shoring initiatives for medical supplies and/or essential drugs have been discussed and are under consideration in different countries, such as the US [43], Spain [44], France [45] and Italy [46].

According to Directorate General for External Policies of the Union [5], reshoring might represent a strategic alternative—to be coupled with the stockpiling alternative—to secure the supply of critical medical products. However, some differences have emerged among medical product subcategories (namely, PPE-like and medical devices) and even within them. As far as PPE-like products are concerned, as clearly shown during the first two waves of the pandemic, it was easy to set up new production lines for face mask production and/or to activate the industrial reconversion of textile and garment industries. However, experts indicate that the question of economic sustainability arises when considering the lower demand for such products after the pandemic. Therefore, it is likely that the production capacity of facial masks in the EU will be sustained only through ad hoc industrial policies, as in the case of public procurement, paying attention to sustainability issues (e.g., CO_2_ emissions due to long-distance transport and/or the use of sustainable materials). In contrast, relocating the production of inspection gloves in the EU or neighboring countries is expected to be highly unlikely, since the concentration of production activities in Malaysia is supported by several comparative advantages (access to raw materials, low labor and power costs, less restricted environmental legislation). Finally, reshoring critical “medical devices” appears easier since production capacity is largely available within EU countries, and technical knowhow is still within European plant departments. However, the real cause of shortages is the dependence on GVCs for critical components, for which a stockpiling approach is easier to implement [5, 19].

### The public national health care system in Spain (MSSSI) [47]

The Spanish National Health System (Sistema Nacional de Salud, SNS) is a universal national health system based on the principles of universality, free access, equity, and financing fairness, and is primarily funded by taxes. The health competencies are transferred to the country’s 17 autonomous communities. The SNS is organized at two levels: national and regional. At the national level, the chief role of the Spanish Ministry of Health is to exercise responsibility for strategic areas such as (i) coordination of the regional systems through a collegiate body; (ii) basic health legislation by publishing regulations, laws, and standards that are mandatory throughout the SNS; (iii) the definition of the basic services portfolio of the SNS; (iv) pharmaceutical policy; and (v) undergraduate and graduate health care education [48]. Health competencies were transferred to the regions between 1981 and 2001. Thus, regional health departments are in charge of the health authority, health care delivery, regulation, planning, budgeting, and third-party payment. In practice, regional systems are autonomous, and there is little tradition of coordination among them [49]. Responsibilities about sourcing and purchasing medicines and medical supplies are delegated to regional health care systems, which are allowed to buy only certified products from approved manufacturers. European regulations determine the technical requirements for products (either medicines, medical supplies, medical equipment, or other) or manufacturers. European regulations for medical supplies are established by the Official Journal of the European Union [50], and are adopted at the national level in all EU countries, being the Ministry of Health and Social Policy in Spain [51].

In Spain, the only body designated by the Ministry of Health and Social Services, and Equality to evaluate medical products is the AEMPS (Agencia Española de Medicamentos y Productos Sanitarios, or the Spanish Agency of Medicines and Medical Products, notified body #0318), which performs these evaluation functions through the Health Products Certification Division (notified organism), and provides authorization within the country, establishing the PVL (Precio de Venta de Laboratorio, or selling price for the laboratory) and the selling price for the SNS. The current regulation in Spain regarding medical aspects establishes: the classification of products; the requirements applicable to essentials and conformity assessment procedures; and the requirements applicable to manufacturing, importing, sterilization, grouping, distribution, commercialization, and sale, adapted to Spanish territory. Medical products are classified into classes I, IIa, IIb, and III, depending on their risk [51].

In recent decades, national governments have implemented several interventions to reduce health care costs [52–58]. Such policies have also been implemented in Spain [59, 60]. The Royal Decree Law 16/2012 in Spain [61] presented a set of urgent measures to guarantee the sustainability of the SNS and to improve the quality and safety of services with the following objectives: (i) increasing financial resources for the system; (ii) controlling expenditures; and (iii) increasing efficiency. In this regard, Gallo and Gené-Badia [60] classified these measures as (i) policies intended to change the level of contributions to publicly financed health care; (ii) policies intended to affect the volume and quality of publicly financed health care; and (iii) policies intended to affect costs.

When considering the purchasing process within the health care industry, it is necessary to consider that managers’ aim to balance efficiency and effectiveness to improve performance entails unique challenges. More specifically, managers have to simultaneously cope with the need to control costs while ensuring exceedingly high-quality patient care. Without such high-quality care, managers risk the nearly immeasurable cost of lost life [62]. Consequently, during a public health crisis, supply strategies and policies became even more complex and critical.

In the case of the Spanish health care system, purchasing decisions can be made at the national or regional health care level or at the hospital level. Purchase orders to suppliers are usually sent by purchasing managers at the hospital level. Purchase decisions at hospital levels are normally made by an internal committee composed of the purchasing manager and people with expertise in economics, pharmacology, or management. Products that could be purchased at the hospital level are classified as medicines/drugs, medical supplies, medical equipment, or other. PPE-like are included in medical supplies. Purchasing decisions at the hospital level must be in compliance with contract laws and regulations that establish that suppliers must follow a qualification process before they can be selected, and contracts are signed. The qualification process refers to a series of technical and economic aspects that the company and the product itself must comply with.

Once products have been certified, they can be offered for purchase by the SNS. The purchase of medical supplies takes place through public bidding, which is regulated by the Public Sector Contract Law (Ley de Contratos del Sector Público, 9/2017). There are different types of procedures for tendering or contracting supplies: open tender (any company that meets the minimum requirements can access the tender), restricted (only certain companies with certain characteristics), or negotiated (with specific companies, e.g., for exclusivity reasons). The most common procedure is open tender. The requirements to be met are then set forth in the technical specifications within each contract. These technical characteristics do not have to be the same in all contracts. For the same medical supply, two hospitals might specify different minimum technical requirements. If a material meets the minimum technical requirements, the evaluation phase of technical offers begins; then other aspects or criteria (previously laid out in the contract specifications) come into play, such as economics, delivery times, or services. In general, the “price” criterion has greater weight in the award; in addition, delivery time and warranty period are highly valued.

The outcome of a tender is the award of the purchase of that product at a price and a quantity over a period of time. The bidding body negotiates the purchase of the products for all the centers included in the framework agreement. The bidding body could be a hospital (in that case, purchases refer only to that hospital), a regional purchasing platform (purchases refer to all the hospitals within this region), or a national purchasing platform (purchases refer to all the hospitals within the country).

There are different types of centralized purchasing platforms. Based on geography, there is (i) a national platform that negotiates contracts for all Spanish hospitals; and there are (ii) regional platforms that negotiate contracts for all hospitals within the region. Based on the central purchasing model, platforms could (i) tender and award the purchase of the products, and then its hospitals would purchase accordingly; or (ii) centralize contracting, purchasing, and/or logistics. Regional platforms are only available in some regions, such as Andalucía, Baleares, Cataluña, Galicia, Murcia, and País Vasco. Other regions, such as Madrid and Castilla La Mancha, do not have centralized purchasing.

## Methodology

The research questions that govern this paper refer to the analysis of how the scarcity of PPE-like during the COVID-19 pandemic has influenced decision-makers’ behavior of supply management in the health care industry. We designed the research following a qualitative, explorative approach based on in-depth, semi-structured interviews with key informants, as well as informal interviews for triangulation. We adopted a case study methodology [63–66], particularly the case study of the buyer’s perspective in the Spanish health care industry.

We performed the design of the interview process (script preparation, planning, selecting interviewees, carrying out interviews, transcription, and coding) according to Kvale [67] and Vallés [68]. We prepared an interview script based on the literature review, although we reformulated the questions and discussion topics into a language suitable for the interviewees to understand. We designed the script structure as follows: (i) an introduction of the research team; (ii) aim and scope of the research project; (iii) the purchasing process in the Spanish health care system and in the interlocutors’ hospital; (iv) procurement and decisions regarding suppliers; (v) offshoring/reshoring; (vi) the impact of COVID-19; (vii) buying PPE-like before COVID-19; (viii) buying PPE-like during COVID-19; (ix) PPE-like sourcing perspectives after COVID-19; and (x) the closing of the interview.

We selected three public hospitals (called A, B and C for confidentiality reasons) located in three different regions of the Spanish SNS. Then, we held four interviews with personnel from these hospitals between January and March 2021. We chose the four key informants to consider the different perspectives of actors involved in the purchasing process. We held two interviews with staff from hospital C, with the aim of enriching the information from the perspectives of purchasing and contract management. Interviewees were people with management responsibilities in the purchasing process, and with more than 10 years of experience. Table 2 summarizes the in-depth semi-structured interviews. For the interviews, we strictly followed a research protocol, where after introducing the research project, we asked specific questions from the interview script. This allowed the interviewees to provide concise or general answers, as they preferred, and encouraged them to respond more freely, bringing new issues to the discussion on the research topic. Moreover, new questions could arise for clarification of comments or answers.

**Table 2:** In-depth semi-structured interviews

Interviewee	Job position	Experience	Date	Duration	Hospital
Int 1	Pharmacy	+ 20 years	January 2021	74 min	A
Int 2	Economics	+ 20 years	February 2021	56 min	B
Int 3	Purchasing	+ 10 years	March 2021	32 min	C
Int 4	Contracts	+ 10 years	March 2021	25 min	C

Source: Designed by authors

With the aim of triangulating and corroborating the context of the gathered information, we took other stakeholders and economic actors into account and held informal interviews. The objective was to better describe the landscape and to understand the legal, economic, and technological context of the industry and sector in Spain. We chose participants for the informal interviews based on the following criteria: (i) at least 10 years of experience in the health care industry in Spain; (ii) experience in management positions in consulting firms and/or health care organizations; and (iii) no connection to or relationship with the three hospitals. Thus, we held four additional, informal, non-structured interviews with industry experts (who offered entrepreneurial, association, industry, and firm-level perspectives) with the goal of validating the context information retrieved from the in-depth interviews. We conducted informal interviews using the same script as in-depth interviews for the main topics, but we did not consider very specific questions (when they were related to specific hospital practices). We did not record or transcribe the informal interviews. Instead, notes were taken by one of the researchers during the interviews. Table 3 summarizes the informal interviews.

**Table 3:** Informal interviews

Interviewee	Job position	Experience	Date	Duration
Int 5	Industry expert	+ 20 years	March 2021	60 min
Int 6	Industry expert	+ 20 years	March 2021	60 min
Int 7	Industry expert	+ 12 years	February 2021	30 min
Int 8	Industry expert	+ 14 years	February 2021	40 min

Source: Designed by authors

Next, we explored, synthesized, and coded the information from the transcriptions by following a content analysis technique; this led to exhaustion in terms of clarity and data saturation [67, 69, 70]. Coding consisted of extracting the concepts of the research data and examining them in terms of their dimensions and properties [69]. We coded the interviews following a double approach: first deductive, then inductive. We started the coding process deductively by extracting the analysis categories from the interview script and the theoretical framework. Once we transcribed the interviews, we continued the coding process inductively. A detailed and precise reading of all materials (transcriptions and notes) allowed us to identify the definitive categories, which are summarized in Table 4. We completed the codification process with the reading of the informal interviews and notes, from which we derived ideas, interpretations, and nuances. This helped us to support or question the information previously obtained, providing more solidity to our findings. When we completed the coding process, we conducted a categorical content analysis, aiming for interpretation.

**Table 4:** Categories after coding the interviews

Categories	Before COVID-19	During COVID-19	After COVID-19 (expected)
Spanish NHS:			
Regulations, protocols	**X**	**X**	**X**
Changes to regulations		**X**	**X**
Supplier selection:			
Drivers, compliance, price, quality, location	**X**	**X**	**X**
Procurement process at hospital:			
Inventory management	**X**	**X**	**X**
Scarcity/availability	**X**	**X**	**X**
Prices, quality	**X**	**X**	**X**
Disruptions in PPE-like sourcing	**X**	**X**	
Strategies & policies in the post COVID-19 era			
Coordination at different levels (national, regional, hospital)			**X**
Protocols to find new suppliers, to purchase			**X**
Collaboration/innovation			**X**
Reshoring (e.g., backshoring vs. nearshoring)			**X**
Stockpiling			**X**

Source: Designed by authors

## Findings

Our paper has two main research aims, the first of which regards the impact of the scarcity that occurred during the pandemic and impacted the behavior of decision-makers in the PPE-like supply chain at the hospital level in Spain. We will present findings that emerged through in-depth semi-structured interviews in the next two subsections, specifically referring to the phases before and during the COVID-19 pandemic.

Our second research aim regards strategies that should be executed to cope with future PPE-like shortages. We will summarize these findings in the third subsection.

### PPE-like sourcing before COVID-19

When requested to describe the purchasing behavior related to the PPE-like supply before the pandemic, all the interviewed people pointed out that Spanish hospitals were used to obtaining their PPE-like supplies from a reduced number of wholesalers, which also offered other references (on average, approximately 2000). As for the main purchasing criterion being the lower price [61], distributors supplied products manufactured in Asian countries, mostly in China. In this respect, the four industry experts pointed out that the lower cost characterizing Asian products was (at least partially) due to the differences between the environmental legislations in such countries when compared to EU member states. More specifically, the European rules related to the environmental impact of the PPE-like production process make them complex and expensive.

Moreover, our respondents agreed that PPE-like products are considered commodity products, and compliance with technical requirements is rather easy for all distributors; thus, all respondents agreed that the products’ technical quality was not a purchasing criterion when selecting the supplier. The following quotes refer to citations from our interviewees (henceforth, we will refer to respondents using the codes shown in Tables 2 and 3).

“We usually work with distributors, ( …) who source materials mainly from Chinese manufacturers. ( …) PPE-like distributors know how to prepare technical documentation for compliance with technical requirements. Then, [once all distributors meet the technical requirements], the main driver for the supplier decision is the price” (Int 3).

“A much higher importance was given, and instructions were dictated so that the price criterion had much more weight than any other objective criteria [for supplier selection]” (Int 4).

However, all of them recognized that PPE-like supplies manufactured in Asia (although owning CE certificates) were perceived as having lower quality than the few supplies manufactured in Europe or the US. In this respect, the following citations confirm these perceptions, with different levels of certainty.

“The general perception is: the quality is lower in products coming from China” (Int 1).

“For products coming from China, the price is infinitely lower, so the suspicion and perception [of lower quality] exists” (Int 2).

“When comparing a ( …) product versus a face mask manufactured in China, the perception of quality changes, it is worse [for the Chinese]” (Int 3).

More specifically, some respondents reported very few cases of refusal of supplied products due to low technical quality.

“Some articles have been returned to suppliers [because of quality issues] before, during and after the pandemic. ( …) Now, the situation has worsened, maybe because now we handle three times more inventory” (Int 3).

In terms of inventory management, all interviewees declared that safety stocks of PPE-like products were generally too low to reduce inventory costs.

“One of the challenges for hospital management is striking the balance between providing good service and minimizing inventory, as inventory, is a cost” (Int 5).

However, safety stocks were generally sufficient to cover the average delivery time due to the huge availability of products at the distributors. Moreover, as a commodity, in the case of a shortage at a certain wholesaler, the product would be easily received by other distributors. Therefore, PPE-like structures were generally reordered based on an automatic approach activated when the reorder point was reached.

Finally, before COVID-19, the prices of PPE-like products were generally stable and based on long-term agreements with distributors.

### PPE-like sourcing during COVID-19

When considering the first wave of the pandemic (which in Spain took place mainly in March–June 2020), all respondents reported several huge shortages of PPE-like products.

“I had never reported shortages of PPE-like products before the pandemic. (…) However, it happened during the first wave “(Int 3).

“The market cannot produce such a high demand in a short time. So we have had a shortage of ( …) and protective equipment” (Int 2).

This was coupled with continuous and intense price increases higher than 200%. Moreover, distributors pushed hospitals’ purchasing offices to make very quick decisions on placing orders, threatening them to sell the few available PPE-like products to other hospitals willing to pay huge prices. Several respondents made this issue clear with respect to different products:

“[When the demand became high and there was a lack of materials] prices increased significantly; this is the law of supply and demand. Face mask prices rose to €10 during the first wave, and some months later, they dropped to €0.90 each” (Int 1).

“There were huge price variations, high increases during the first wave. ( …) distributors and dealers urged hospitals to accept their prices at those times, saying that if they did not do it, they could sell the goods to other hospitals which would pay for them” (Int 6).

“100% or 200%, [huge price variations], even higher. Look, the glove box price under normal conditions is €4.5. Currently, the glove box is at €10.5 and they have been offered at €12, and not lower than €10 in any case” (Int 2).

“Prices increased. A clear example is nitrile gloves. Gloves in general went up a lot in price; they have been fluctuating a lot. The explanation that the industry gave you was that it was due to the raw material, the nitrile, that there was even a shortage of raw material” (Int 3).

Respondents also reported criticalities in terms of quality issues. For instance, one of the interviewees (Int 3) mentioned the case of a PPE-like lot having the label “Not for medical use,” even if the product imported from China had the CE label. However, according to Int 3 and some of the interviewed industry experts (Int 6 and Int 7), the use of such a label could be explained with the aim of overpassing the Chinese government ban on PPE-like exports for medical use. More specifically, the respondent reported very few cases of refusal of supplied products due to low technical quality.

“Some articles were returned to suppliers [because of quality issues] before, during and after the pandemic. ( …) Now, the situation has worsened, maybe because now we handle three times more inventory” (Int 3).

Moreover, according to all respondents, delivery times become dramatically longer, both for supplies coming from China and those manufactured in the very few European plants available.

“Mobility restrictions and the closing of borders during the first wave provoked shortages of products and significant delays in deliveries” (Int 8).

Finally, all interviewees stated that health care professionals (e.g., doctors and nurses) experienced a lot of anxiety due to PPE-like scarcity, along with fears about becoming infected by COVID-19. This anxiety was made even worse by the need to reuse products due to their shortage.

“When there were shortages of PPE-like products [fear and anxiety], there were some photos ( …) in the ICU of a hospital in ( …) where hospital professionals were wearing plastic garbage bags [as protective equipment] over their clothes, and this was scary” (Int 1).

“Professionals experienced fear and anxiety. In fact, there was reuse of materials” (Int 2).

“The use of partial shields, the helmet of a welder, use of a material more hours than recommended, etc.” (Int 2).

“There was concern on the part of certain groups [of hospital professionals], as well as unions” (Int 3).

When considering solutions implemented to cope with the critical situation, the respondents referred to several solutions implemented at the national, regional, and hospital levels. All respondents pointed out that such actions were executed according to a parallel approach, and recognized that while this approach aimed to maximize the chance of success, the lack of coordination resulted in increasing search and purchasing costs, creating competition between actors and further favoring the few distributors with products to be supplied.

As reported by the interviewees, the scarcity of PPE-like induced the national government to relax purchasing requirements, at least for some categories. In this respect, the qualification procedures for new suppliers were, at least partially, made less stringent.

“Given the shortage, it was necessary to look for alternatives, even using protective materials that were not approved but that could serve as protection” (Int 2).

At the regional level, two different approaches were carried out because of differences in the PPE-like purchasing systems implemented before the pandemic. More specifically, while some regional health care authorities authorized emergency purchasing procedures (especially between March and the end of August 2020), other administrations decided to recentralize the previous decentralized purchasing system. Some organizations also implemented autonomous initiatives directly with foreign intermediates and manufacturers. For instance, a respondent stated:

“Actions were taken [at the regional level], such as sending a plane directly to China to obtain materials” (Int 3).

Finally, we should note the emergence of innovation needs, such as the joint use of one ventilator to simultaneously serve two patients, and the adaptation of Decathlon’s diving masks for use in ventilatory therapy. To cope with these needs, hospitals often worked with “external actors” (mainly manufacturing companies and “makers,” and less so with universities and research centers). Moreover, collaborations took place with other hospitals, both in Spain and abroad. However, such innovations mostly originated from individual initiatives (a bottom-up approach) rather than from the governance level (a top-down approach). For instance, a respondent mentioned:

“Collaboration took place with a local company, which produced plastic covers to be used as face masks” (Int 1).

“The pandemic helped to increase collaboration between hospitals. We created a system to share medicines and other materials among hospitals. ( …). It worked very well, and this system will continue after COVID-19” (Int 1).

### Suggestions for the post COVID-19 era

When requested to suggest that strategies and policies should be implemented in the post-COVID-19 era to make Spanish health care able to cope with future public health crises, all respondents shared the idea of implementing significant changes in the regulatory framework. More specifically, it was recommended to modify the national law regulating public procurement procedures for the health care industry.

“The government announces ( …) the reform of the public health contracting model ( …). Because it has to be much more agile” (Int 4).

Moreover, they pointed out that the national and regional authorities should replace the purchasing criterion based on continuous cost reductions, considering the value of human lives. The interviewees pointed out that the scarcity of PPE-like heavily impacts human life; thus, cost should never be the central decision-making driver.

“The pandemic reinforced the idea that other factors [apart from cost] such as quality or service should be taken into consideration to guarantee the availability of products, at least for critical ones” (Int 6).

A further intervention suggested by all respondents regards regions implementing a decentralized purchasing system earlier; in such a case, a centralized approach at the regional level is preferable.

“I consider a central purchasing office [at the regional level] to be a great idea” (Int 1).

“Centralizing the regional purchasing increases negotiation power” (Int 2).

Moreover, it was suggested that collaborations should be promoted between hospitals and among them and other external actors.

When considering inventory management policies, there was a consensus among respondents about the need to increase the safety stock levels to better manage the risk of PPE-like shortages.

“Safety stocks have increased significantly because consumption has increased a lot, ( …). The pandemic brought about uncertainty and difficulties to make accurate forecasts” (Int 3).

“The stock of PPE-like products in the warehouse has tripled when compared to one year before COVID-19 and now. Therefore, our stock in the warehouse, ( …) especially now that we have just overcome a very high peak of patients, is very high, especially of these materials [PPE-like]” (Int 3).

Finally, interesting findings emerged when considering the portfolio of existing and future suppliers and the locations of their production facilities. First, the respondents suggested enlarging the number of authorized suppliers, especially Spanish and EU suppliers, which were excluded earlier due to their higher prices. In this respect, all but one respondent stated that Spain should reach strategic autonomy in terms of medical devices, including PPE-like devices. According to them, the establishment of new production lines of PPE-like in Spain—or at least in Europe—would mitigate future risks of supply chain disruption, but also improve product quality, since output in Western countries is assumed to be more reliable. Moreover, lead times were reduced, and service levels increased. Finally, the respondents also cited the positive impacts in terms of economic development of the Spanish/EU economic system.

According to the respondents, strategic autonomy by the Spain/EU health care industry in terms of PPE-like product procurement could be achieved by promoting either back-shoring (relocation to the home country of earlier offshored production activities) or near-shoring (relocation to the home region, namely, the EU) strategies. However, in the latter case, the interviewees requested a clear definition of export terms, and the conditions that would be necessary among European countries to avoid what happened during the first wave (March–June 2020) when Germany and France decided to cancel shipments to Spain and Italy.

“Today, there are some initiatives under discussion on how to backshore the production of some items in Spain, in the same way as it is under consideration in other countries like Italy or France” (Int 7).

“At least for some references, critical references, the government should incentivize their production within Spain or Europe, with the aim of guaranteeing availability and good service” (Int 8).

Finally, only one respondent (Int 4) opposed the production of PPE-like products in Spain and/or in other European countries. More specifically, she proposed only increasing the levels of inventories while also implementing stock-pilling facilities.

“In my opinion ( …) we must not prioritize the [manufacturing] location, because I believe that it is discrimination ( …). We might consider higher stocks and shorter lead times, regardless of where [suppliers] are located” (Int 4).

## Discussion

Findings emerged from the in-depth semi-structured interviews, offering interesting insights to shed light on the two research aims discussed in this paper. As far as the first one (the impact of PPE-like product scarcity on the behavior of decision-makers at the hospital level), the evidence shows that the Spanish health care system was not ready to cope with a sudden shortage of such products, since they had always been available in the market thanks to long-term contracts signed with multiproduct distributors. In this regard, a total absence of previous risk assessment for this supply typology is apparent, despite previous pandemics such as Ebola and SARS [6]. An adequate analysis of a previous health crisis would have supported the creation of a more effective PPE-like supply chain. In light of this, it would be developed, ensuring a higher level of supply chain visibility, which has been recognized as a critical element “to enabling critical decision-making accuracy, which will in turn increase the ability of local, state and federal health care and public health decision-makers to respond” [71].

Moreover, the scarcity of PPE-like during the pandemic had a huge impact on the Spanish health care system. It is worth nothing the impact on professionals, who were very worried about becoming infected [11], especially when reuse policies were implemented in their hospitals [21]. This, in turn, seems to have heavily influenced the perception and purchasing behavior of actors involved in the PPE-like supply chain at the hospital level. Therefore, COVID-19 may serve as a trigger for a deep change in the Spanish health care supply decision-making process.

The magnitude and direction of such a change is clearly seen in several issues when comparing the “before COVID-19” and the “during COVID-19” phases. First, before the pandemic, the nearly only purchasing criterion was the price (due to regulatory requirements at the national level, [61]); during the health crisis, the mere availability of PPE-like products became the chief criterion. This induced both hospitals and national/regional administrations to not refer only to supply by traditional distributors, but also to establish direct contacts with manufacturers, often through new intermediaries who suddenly appeared in the market. Therefore, long-term contracts were sharply substituted by spot contracts, with an immediate impact on the product price, which after a long period of stability, rapidly increased by more than 200%. At the same time, adherence to procedures—which was compulsory before the health crisis—was relaxed, especially in terms of suppliers’ qualifications.

Moreover, a generally decentralized purchasing approach sharply evolved toward a mixed approach in which all health care actors (single hospital, regional and national administrations) individually operated in the market to obtain as large a quantity of supplies as possible. While this would have improved the chance of success, it resulted in creating competition and a further rise in the supply price.

However, at least two elements seem to have remained stable before and during the COVID-19 crisis: the reduced attention reserved to product quality, and sustainability issues. In the ex-ante phase, this was explained by the legal obligation to buy products at a lower price; during the first wave of the pandemic, it was due to the impossibility of obtaining the necessary amount of PPE-like products.

When considering the second research aim (i.e., strategies and policies to be implemented in the post COVID-19 era), three main issues become apparent:

- the shift from a supply strategy focused on cost issues to one based on availability and product quality;
- the call for a higher level of centralization of the supply decision-making process;
- the request for a national/EU production base for PPE-like products.

The change in the supply strategy’s aim (from a cost-oriented one to another addressing quality and availability) is consistent with proposals by several authors in the last decade [8, 28, 52, 53, 55–60]. However, it seems that COVID-19 may represent a real trigger role in modifying policy-makers’ mindset, since the demand for an affordable public health care system is emerging in all Western countries [72]. Moreover, some EU national governments (such as Spain, Italy, and France) may redefine their health care policies and finance them with funds from the Next Generation EU program.

At the same time, respondents gave evidence of the negative consequences of a federative approach to the health care system, which may generate a lack of coordination between single regional health care systems [28]. Centralization of purchasing decisions is a particular issue for policy-makers in Spain. Based on our findings, the current federal system—where each region independently manages its health system [49]—seems no longer able to handle the purchasing decision-making process during a health crisis. A consensus and deeper coordination among regions are consequently requested to optimize the availability and costs of PPE-like medical products. In this regard, Meehan et al. [73] showed that the focus on unit price savings creates barriers for cooperation and aggregated procurement. Finally, we should note the role of information technologies (such as digital supply platforms) in supporting the centralization of the medical product purchasing process. These technologies can also provide a higher degree of visibility along the supply chain, which is critical, especially during health crises [71].

The third main finding arising from the collected data regards the request of either a Spanish or EU production base for PPE-like products to ensure what has been defined as “strategic autonomy” in terms of medical products [5]. Thus, back-shoring or near-shoring may represent an appropriate alternative to supply from low-cost Asian countries, consistent with the most recent extant literature [5, 7, 40, 42]. However, we must take into account that “even something as simple as an N95 mask uses—according to the label on the box—‘globally sourced materials’” [7], like melt blown (a type of non-woven polypropylene) and nose clips. The large majority of the production capacity of these two raw materials is concentrated in China, thereby maintaining the risk of supply chain disruption, even in the case of face mask production located in European countries.

Moreover, only one respondent recommended relying on stockpiles to ensure PPE-like behavior in the case of a future health crisis. As such, Feinmann [7] pointed out that after the 2009 H1N1 pandemic (SARS), the US strategic National Stockpile distributed facial masks to federal states and localities, but it did not replenish stocks after that crisis [74]. Similar evidence arose in the case of the UK’s emergency stockpile, created in 2006 as part of the national pandemic influenza preparedness program. It included, among others, gloves and respirator masks but not gowns, despite a specific recommendation from the New and Emerging Respiratory Virus Threats Advisory Group (Nervtag). Moreover, the BBC found that at least two-thirds of masks included in the original 2009 procurement list were no longer available. Finally, the management of the stockpile was outsourced; the current management company was in the process of being sold [7]. Once more, we can see that Western countries’ health care authorities seem to have underestimated the role of PPE-like products in the health care crisis [6].

The matter of sustainability deserves our attention, since respondents never mentioned it as a purchasing criterion, even in the post COVID-19 era. This is even more unexpected when the use of single-use plastic PPE-like material is considered a growing challenge to environmental sustainability. In this regard, Rowan and Laffey [75] made a huge effort to promote product innovations that allow for the reuse of PPE-like products. Moreover, they state that it will also be necessary to build trust and acceptance of these eco-friendly products among health care workers.

## Conclusions

To the best of our knowledge, our paper represents a first attempt to evaluate COVID-19’s impact on the behavior of decision-makers in the PPE-like supply chain at the hospital level.

The findings from the in-depth, semi-structured interviews with key informants provide very useful insights for practitioners and policy-makers involved in the health care industry. As far as the former is concerned, they should carefully evaluate how to make their supply and purchasing policies more effective after executing a careful risk. At the same time, policy-makers should promote policies aiming to centralize the purchasing decision-making process (at least in the case of a health crisis) and to improve coordination among hospitals and regional health care systems. Finally, European national governments and EU institutions should carefully reflect on how to reach “strategic autonomy” in this context [5, 40, 76].

This paper is bound by certain limitations. First, it is based on a few Spanish hospitals, which is consistent with its exploratory nature. However, future research should widen the scope of analysis and implement comparisons at the international level to consider institutional constraints (if any). Moreover, we only focused on a specific subcategory of medical products, namely, PPE-like products. Future research should also consider other health care products, including medical devices and drugs. Moreover, since we conducted our study while the COVID-19 pandemic is still ongoing, it would be useful for future research to examine long-term effects on purchasing strategies and policies.

## Data Availability

Data sharing is not applicable to this article, as no datasets were generated or analyzed during the current study. Transcripts from interviews are available from the corresponding author on reasonable request.

## Abbreviations

AEMPS: Spanish Agency of Medicines and Medical Products
GVC: Global Value Chain
EU: European Union
H1N1: Hemagglutinin Type 1 and Neuraminidase Type 1
MERS: Middle East Respiratory Syndrome
MSPS: Ministry of Health and Social Policy
PPE: Personal Protective Equipment
PVL: Laboratory Selling Price
SARS: Severe Acute Respiratory Syndrome
COVID-19: Coronavirus disease 2019
SNS: National Health System
WHO: World Health Organization

## References

[1] World Health Organization, WHO Director-General's opening remarks at the media briefing on COVID-19 - 11 March 2020a, online access on: https://www.who.int/director-general/speeches/detail/who-director-general-s-opening-remarks-at-the-media-briefing-on-covid-19%2D%2D-11-march-2020 (Accessed on 21/05/2021.

[2] Gereffi G (2020). What does the COVID-19 pandemic teach us about global value chains? The case of medical supplies. J Int Bus Pol.

[3] OECD. The face mask global value chain in the COVID-19 outbreak: Evidence and policy lessons, OECD Policy Responses to Coronavirus (COVID-19), 2020. The face mask global value chain in the COVID-19 outbreak: Evidence and policy lessons (oecd.org).

[4] Hamrick D, Bamber P (2019). Pakistan in the medical device global value chain [internet]. Duke.Edu.

[5] Directorate General for External Policies of the Union – Policy Department (2021). Post Covid-19 options for reshoring production back to Europe in a globalized economy, European Union.

[6] Patel A, D’Alessandro MM, Ireland KJ, Greg Burel W, Wencil EB, Rasmussen SA (2017). Personal protective equipment supply chain: Lessons learned from recent public health emergency responses, Health Security.

[7] Feinmann, J. PPE: what now for the global supply chain? British Medical Journal (BMJ): first published as 10.1136/bmj.m1910 on 15 May 2020. online access on http://www.bmj.com/ (Accessed on 01/5/2021). 10.1136/bmj.m1910 32414747

[8] Cohen J, van der M RY (2020). Contributing factors to personal protective equipment shortages during the COVID-19 pandemic. Prev Med.

[9] Pampillón Olmedo R, Pampillón AR (2017). Spain, is the expenditure on R&D a determining factor in its external competitiveness?. Harvard Deusto Business Research.

[10] Dai T, Bai G, Anderson GF (2020). PPE supply chain needs data transparency and stress testing. J Gen Intern Med.

[11] Urooj U, Ansari A, Siraj A, Khan S, Tariq H (2020). Expectations, Fears and Perceptions of doctors during Covid-19 Pandemic. Pak J Med Sci.

[12] Padilla J, Mendez (2021). Insertion of Emerging Countries Exports in Global Supply Chains. Harvard Deusto Business Research.

[13] Blanco-Donoso LM, Moreno-Jiménez J, Amutio A, Gallego-Alberto L, Moreno-Jiménez B, Garrosa E (2021). Stressors, job resources, fear of contagion, and secondary traumatic stress among nursing home workers in face of the COVID-19: the case of Spain. J Appl Gerontol.

[14] Perspectives HJ (2020). COVID-19 and PPE in context: an interview with China. J Public Health.

[15] Plana D, Tian E, Cramer AK, Yang H, Carmack MM, Sinha MS, Bourgeois FT, Yu SH, Masse P, Boyer J, Kim M, Mo J, LeBoeuf NR, Li J, and Sorger, PK. Assessing the quality of nontraditional N95 filtering face-piece respirators available during the COVID-19 pandemic. medRxiv 2020, 20161968; doi: 10.1101/2020.07.25.20161968. 10.1186/s12879-021-06008-8 PMC8319695 34325673

[16] Hackenbroich J, Shapiro J, Varma T (2020). Health sovereignty: how to build a resilient European response to pandemic. European Council on Foreign Relations, Policy Brief.

[17] World Health Organization. Coronavirus disease 2019 (COVID-19): Weekly epidemiological update. – 06 September 2020b, online access on: https://www.who.int/docs/default-source/coronaviruse/situation-reports/20200907-weekly-epi-update-4.pdf?sfvrsn=f5f607ee_2 (Accessed on 06/07/2021).

[18] Europe MT (2020). The European medical technology industry in figures 2020. MedTech.

[19] Grumiller J, Grohs H (2021). Increasing security of supply for critical medical pharmaceutical goods in the EU: lessons from Covid-19 pandemic. OFSE Austrian Foundation for Development Research.

[20] Yazid NM, Yatim AHMNKEA (2014). Positioning of the Malaysian rubber gloves industry. MRB Rubber Technology Developments.

[21] Ranney M, Griffeth V, Jha A (2020). Critical supply shortages – the need for ventilators and personal protective equipment during the Covid-19 pandemic. N Engl J Med.

[22] Netland T (2020). A better answer to the ventilator shortage as the pandemic rages on.

[23] Chellamani KP, Veerasubramanian D, Balaji RSV (2013). Surgical face masks: manufacturing methods and classification. J Acad Ind Res (JAIR).

[24] Pecchia L, Piaggio D, Maccaro A, Formisano C, Iadanza E (2020). The inadequacy of regulatory frameworks in time of crisis and in low-resource settings: personal protective equipment and COVID-19″. Heal Technol.

[25] Shingal A, Agarwal P (2020). Global value chain responses to previous health shocks: Lessons for Covid-19.

[26] Bown C. China should export more medical gear to Battle COVID 19. Piie. com, 2020. Available from: https://www.piie.com/blogs/trade-and-investment-policy-watch/china-should-export-more-medical-gear-battle-covid-19#:~:text=As%20emotionally%20satisfying%20as%20it,now%20in%20short%20supply%20globally.

[27] Asian Development Bank Global Shortage of Personal Protective Equipment amid COVID-19: Supply Chains, Bottlenecks and Policy Implications, 2020. ADB Briefs 130. https://www.adb.org/sites/default/files/publication/579121/ppe-covid-19-supply-chains-bottlenecks-policy.pdf

[28] Sharma A, Adhikary A, Bikash BS (2020). Covid-19’s impact on supply chain decisions: strategic insights for NASDAQ 100 firms using twitter data. J Bus Res.

[29] European Commission (2020). COVID-19: Commission creates first ever rescEU stockpile of medical equipment, Press corner.

[30] Bono F, Sevillano E (2020). With hospitals facing shortages, Spanish shoemakers start sewing face masks to fight coronavirus crisis [Internet]. Elpais.com.

[31] The Local. What is Spain doing about its face mask shortage?”, Date 26 March 2020. Available online at: https://www.thelocal.es/20200326/spains-face-mask-shortage-sparks-cottage/ (Accessed on 06/05/2021).

[32] Cadena Ser. Las bodegas y destilerías también se ponen al servicio de la lucha contra el coronavirus. Available from: https://cadenaser.com/ser/2020/03/20/gastro/1584716104_150768.html. (Accessed on 06/05/2021).

[33] El Economista (2020). Francia transformará excedentes de vino en desinfectante de manos y etanol.

[34] Forbes (2020). Elaboran gel antibacterial con vino destilado en Baja California. Forbes Magazine.

[35] Gómez FA (2020). “El vino sin vender en Italia se convierte en gel desinfectante contra el coronavirus”. ABC Sociedad.

[36] Maestelli M (2020). Dalla grappa agli igienizzanti, l’alcol delle distillerie contro il coronavirus. Sole 24 Ore.

[37] France24. Ferrari helps design new low-cost ventilator in coronavirus battle [Internet]. France 24. 2020. Available from: https://www.france24.com/en/20200513-ferrari-helps-design-new-low-cost-ventilator-in-coronavirus-battle. (Accessed on 06/05/2021).

[38] Mezcua U (2020). La Agencia Española de Medicamentos y Productos Sanitarios autoriza los respiradores de Seat. ABC Motor.

[39] UMA. El respirador artificial desarrollado por investigadores de la UMA ya cuenta con la autorización de la Agencia del Medicamento”. Universidad de Málaga, Date 02 April 2020. Available online at: https://www.uma.es/sala-de-prensa/noticias/el-respirador-artificial-desarrollado-por-investigadores-de-la-uma-ya-cuenta-con-la-aut%2D%2D/ (Accessed on 06/05/2021).

[40] Barbieri P, Boffelli A, Elia S, Fratocchi L, Kalchschmidt M, Samson D (2020). What can we learn about reshoring after Covid-19?. Oper Manag Res.

[41] Fratocchi L, Di Mauro C, Barbieri P, Nassimbeni G, Zanoni A (2014). When manufacturing moves back: concepts and questions. J Purch Supply Manag.

[42] UNCTAD, World Investment Report (2020). International production beyond the pandemic.

[43] Roehr B (2020). Bringing drug production home: how the US is rebuilding the drug supply chain after covid-19. BMJ.

[44] Farmaindustria (2021). Farmaindustria presenta al Gobierno un proyecto tractor para impulsar la fabricación de medicamentos esenciales - Farmaindustria.es.

[45] Ministère de l’économie, de finance et de la relance (2020). Le plan d’action pour la relocalisation des industries de santé en France.

[46] Advanced life science in Italy. Progetto per il reshoring di farmaci e principi attivi farmaceutici in Italia, 2020. Progetto per il reshoring di farmaci e principi attivi farmaceutici in Italia ~ Cluster Alisei

[47] Ministerio de Sanidad, Servicios Sociales e Igualdad (MSSSI), 2012. Sistema Nacional de Salud. España, Madrid; 2012. Available online at: www.mscbs.es. (Accessed on 15/07/2021).

[48] Cabo SJ (2010). Gestión sanitaria integral: pública y privada.

[49] Bernal-Delgado E, García-Armesto S, Oliva J, Sánchez Martínez FI, Repullo JR, Peña-Longobardo LM, Manuel RL, Hernández-Quevedo C (2018). Spain: health system review. Health Systems in Transition.

[50] Official Journal of the European Union (OJEU). Regulation (EU) 2017/745 of the European Parliament and of the Council of 5 April 2017 on medical devices, amending Directive 2001/83/EC, Regulation (EC) No 178/2002 and Regulation (EC) No 1223/2009 and repealing Council Directives 90/385/EEC and 93/42/EEC.

[51] Ministerio de Sanidad y Política Social (MSPS). Real Decreto 1591/2009, de 16 de octubre, por el que se regulan los productos sanitarios. BOE-A-2009-17606, BOE núm. 268, de 6 de noviembre de 2009, páginas 92708 a 92778 (71 págs.).

[52] Goldman DP, Joyce GF, Zheng Y (2007). Prescription drug cost sharing: associations with medication and medical utilization and spending and health. JAMA.

[53] Stuckler D, Basu S, McKee M, Suhrcke M (2010). Responding to the economic crisis: a primer for public health professionals. J Public Health.

[54] Stuckler D, Basu S, Suhrcke M, Coutts A, McKee M (2009). The public health effect of economic crises and alternative policy responses in Europe: an empirical analysis. Lancet.

[55] Kentikelenis A, Karanikolos M, Papanicolas I, Basu S, McKee M, Stuckler D (2011). Health effects of financial crisis: omens of a Greek tragedy. Lancet.

[56] Fahy N (2012). Who is shaping the future of European health systems?. BMJ..

[57] Giulio de Belvisa A, Ferrè F, Specchia ML, Valerio L, Fattore G, Ricciardi W (2012). The financial crisis in Italy: implications for the healthcare sector. Health Policy.

[58] Kastanioti C, Kontodimopoulos N, Stasinopoulos D, Kapetaneas N, Polyzos N (2012). Public procurement of health technologies in Greece in an era of economic crisis. Health Policy.

[59] Gené-Badia J, Gallo P, Hernández-Quevedoc C, García-Armestod S (2012). Spanish health care cuts: penny wise and pound foolish?. Health Policy.

[60] Gallo P, Gené-Badia J (2013). Cuts drive health system reforms in Spain. Health Policy.

[61] B O E. Royal Decree Law 16/2012 Urgent measures to guarantee the sustainability of the National Health System and improve the quality and safety of services. Real Decreto-ley 16/2012, de 20 de abril, de medidas urgentes para garantizar la sostenibilidad del Sistema Nacional de Salud y mejorar la calidad y seguridad de sus prestaciones. Boletín Oficial del Estado. 2012;N. 98.

[62] Kros JF, Kirchoff JF, Falasca M (2019). The impact of buyer-supplier relationship quality and information management on industrial vending machine benefits in the healthcare industry. J Purch Supply Manage.

[63] Eisenhardt KM (1989). Building theories from case study research. Acad Manag Rev.

[64] Voss C, Sikriktsis N, Frohlic M (2002). Case research in operations management. Int J Oper Prod Manag.

[65] Eisenhardt KM, Graebner ME (2007). Theory building from cases: opportunities and challenges. Acad Manag J.

[66] Yin RK. Case study research and applications: design and methods. 6th ed. Los Angeles: Sage Publications; 2017.

[67] Interviews KS (1996). An introduction to qualitative research interviewing.

[68] Vallés M (2002). Entrevistas Cualitativas. Cuadernos Metodológicos.

[69] Rubin HJ, Rubin L (1995). Qualitati ve interviewing. The art of hearing data.

[70] Corbin J, Strauss A (2008). Basics of qualitative research.

[71] Finkenstadt DJ, Handfield R (2021). Blurry vision: supply chain visibility for personal protective equipment during COVID-19. J Purch Supply Manag.

[72] King JS (2020). Covid-19 and the Need for Health Care Reform. N Engl J Med.

[73] Meehan J, Menzies L, Michaelides R (2017). The long shadow of public policy; barriers to a value-based approach in healthcare procurement. J Purch Supply Manag.

[74] Estes, A.C. Trump’s strategic National Stockpile looks empty amid ventilator, mask, and PPE shortage. Vox 2020 7. https://www.vox.com/recode/2020/4/3/21206170/us-emergency-stockpile-jared-kushner-almost-empty-coronavirus-medical-supplies-ventilators

[75] Rowan NJ, Laffey JG (2021). Unlocking the surge in demand for personal and protective equipment (PPE) and improvised face coverings arising from coronavirus disease (COVID-19) pandemic – implications for efficacy, re-use and sustainable waste management. Sci Total Environ.

[76] Elia S, Fratocchi L, Barbieri P, Boffelli A, Kalchschmidt M. Post-COVID-19 reconfiguration from global to domestic and regional value chains: the role of industrial policies”. Transnational Corporations Journal. 2021;28:2.

